# A Novel Role of Dapagliflozin in Mitigation of Acetic Acid-Induced Ulcerative Colitis by Modulation of Monocyte Chemoattractant Protein 1 (MCP-1)/Nuclear Factor-Kappa B (NF-κB)/Interleukin-18 (IL-18)

**DOI:** 10.3390/biomedicines10010040

**Published:** 2021-12-25

**Authors:** Mohamed Kh. ElMahdy, Samar A. Antar, Ehab Kotb Elmahallawy, Walied Abdo, Hayfa Hussin Ali Hijazy, Ashraf Albrakati, Ahmed E. Khodir

**Affiliations:** 1 Department of Pharmacology, Faculty of Pharmacy, Horus University-Egypt, New Damietta 34518, Egypt; mkhalil@horus.edu.eg (M.K.E.); santar@horus.edu.eg (S.A.A.); akhodir@horus.edu.eg (A.E.K.); 2 Department of Zoonoses, Faculty of Veterinary Medicine, Sohag University, Sohag 82524, Egypt; 3 Department of Veterinary Pathology, Faculty of Veterinary Medicine, Kafrelsheikh University, Kafrelsheikh 33516, Egypt; 4 Department of Family Education, Faculty of Education, Umm Al-Qura University, Makka Al-Mukarama 21955, Saudi Arabia; hhhijazi@uqu.edu.sa; 5 Department of Human Anatomy, College of Medicine, Taif University, P.O. Box 11099, Taif 21944, Saudi Arabia; a.albrakati@tu.edu.sa

**Keywords:** Dapagliflozin, ulcerative colitis, anti-apoptotic, anti-inflammatory-inflammasome-MCP1-IL-18

## Abstract

Colon illnesses, particularly ulcerative colitis, are considered a major cause of death in both men and women around the world. The present study investigated the underlying molecular mechanisms for the potential anti-inflammatory effect of Dapagliflozin (DAPA) against ulcerative colitis (UC) induced by intracolonic instillation of 3% *v*/*v* acetic acid (AA). DAPA was administered to rats (1 mg/kg, orally) for two weeks during the treatment regimen. Interestingly, compared to the normal group, a marked increase in the index of colon/body weight, colon weight/colon length ratio, serum lactate dehydrogenase (LDH), and C-reactive protein (CRP), besides decrease in the serum total antioxidant capacity (TAC), were reported in the AA control group (*p* ˂ 0.05). Elevation in colon monocyte chemoattractant protein (MCP1), Interleukin 18 (IL-18), and inflammasome contents were also reported in the AA control group in comparison with the normal group. In addition, colon-specimen immunohistochemical staining revealed increased expression of nuclear factor-kappa B (NF-κB) and Caspase-3 with histopathological changes. Moreover, DAPA significantly (*p* ˂ 0.05) reduced the colon/body weight index, colon weight/colon length ratio, clinical evaluation, and macroscopic scoring of UC, and preserved the histopathological architecture of tissues. The inflammatory biomarkers, including colon MCP1, IL-18, inflammasome, Caspase-3, and NF-κB, were suppressed following DAPA treatment and oxidants/antioxidants hemostasis was also restored. Collectively, the present data demonstrate that DAPA represents an attractive approach to ameliorating ulcerative colitis through inhibiting MCP1/NF-κB/IL-18 pathways, thus preserving colon function. Antioxidant, anti-inflammatory, and anti-apoptotic properties of DAPA are implicated in its observed therapeutic benefits.

## 1. Introduction

Ulcerative colitis (UC) is considered one of the chronic idiopathic inflammatory disorders of colonic mucosa that influences the rectum besides other parts of the colon, resulting in a series of symptoms of abdominal pain, rectal urgency, and bloody diarrhea [1]. It should be stressed that this chronic gastrointestinal disorder affects men and women equally and it is also correlated with inheritable genetic traits [2]. There is high incidence of colorectal cancer in patients with UC [3]. There is no exact etiology of the incidence of UC, but multiple factors are included in the pathogenesis of it. Oxidative stress is an essential factor involved in the pathophysiology of disease [4]. Oxidative stress is induced as a result of inflammation and results in DNA damage and carcinogenesis in local and global sites [5]. There is infiltration of leukocytes, neutrophils, and macrophages in the colon, leading to augmented production of pro-oxidant molecules [6], resulting in increased levels of myeloperoxidase (MPO) induced by cytokines with generation of reactive oxygen species (ROS) [7]. Patients with UC suffer from superficial mucosal inflammation without granulomata but those patients exhibit diffuse infiltration of inflammatory cells, reduction in mucus-secreting goblet cells, basal plasmacytosis, and crypt architectural distortion [8]. It seems that there is genetic association with occurrence of UC, which is represented by its development in patients with impaired immune responses against intraluminal antigens, and therefore it has been listed among the immune-mediated disorders [9]. Taken into account, the mucosal changes reported in inflammatory bowel disease (IBD) are expressed by ulcerative lesions which are accompanied by infiltration of various inflammatory cells including macrophages, plasma cells, T lymphocytes, and neutrophils [10]. There are several mechanisms that illustrate the activation and chemotaxis of inflammatory cells and inflammatory mediators. The intensity of inflammation in UC is determined by production of growth factors and proinflammatory cytokines inside the mucosa, which is further controlled by processes of cellular recruitment, such as overexpression of vascular adhesion molecules and chemokine expression [11]. 

Inflammation is involved in the pathogenesis of ulcerative colitis [12]. Interleukin (IL) and tumor necrosis factor-alpha (TNF-α) are among the proinflammatory molecules that are regulated by the nuclear factor-kappa B (NF-κB) family of transcription factors [13]. More importantly, NF-κB is considered a key signaling molecule in the inflammatory process and it promotes the expression and release of pro-inflammatory cytokines, leading to a cascade of inflammatory responses and mucosal injury [14,15]. In addition, NF-κB activates inflammasomes, which are an essential part of the innate immune system, resulting in activation of nucleotide binding and oligomerization domain-like receptor family pyrin domain-containing 3 (NLRP3) complex. However, its dysregulation contributes to several metabolic disorders, such as type-2 diabetes, atherosclerosis, and autoinflammatory disorders such as UC [16]. The activation of inflammasome also targets NF-κB translocation-dependent expression of NLRP3 and formation of mature IL-1β, and this inflammatory cascade is crucial for the pathogenesis of UC [17]. Dapagliflozin (DAPA), a sodium glucose co-transporter 2 inhibitor (SGLT2-I), has proven to be an effective hyperglycemic suppressor due to its role in inhibiting the reabsorption of 30–50% of the glucose filtered by the kidney, besides its role in improvement of insulin resistance [18]. Furthermore, three months of treatment with DAPA has resulted in marked increase in lipolysis rate and insulin sensitivity, which result from its role in increasing levels of the lipid-mobilizing adipokine zinc-alpha2-glycoprotein (ZAG) in blood [19]. Some previous studies have also shown that inhibition of SGLT2 might suppress the expression of inflammatory cytokine and inflammasome activation, besides its role in protecting the kidney from inflammation [20]. However, the mechanisms underlying the colon-protective effect of SGLT2 inhibitors remain unclear. Given the above information, the present work aimed to investigate the possible protective effect and the pathomorphological features of Dapagliflozin on ulcerative colitis in a rat model induced by acetic acid, combined with assessment of the antioxidant status and tissue pro- and anti-inflammatory mediators following treatment.

## 2. Materials and Methods

### 2.1. Ethical Considerations

The study was approved by the Research, Publication and Ethics Committee of the Faculty of Veterinary Medicine, Kafrelsheikh University, Egypt with approval code number KFS-2020/03. 

### 2.2. Drugs

Dapagliflozin (10 mg tablets) was purchased and provided by (AstraZeneca pharmaceutical company, Cairo, Egypt). Before its usage, the drug was suspended in 0.5% carboxymethylcellulose (0.5%). Acetic acid was also purchased from (Chema jet chemical company, Alexandria, Egypt).

### 2.3. Animals and Experimental Design

In the present work, a total of 40 male Sprague–Dawley rats, weighing 180–220 g, were used for the experiment and were obtained from the Modern Veterinary Office for Laboratory Animals (Cairo, Egypt). Throughout the experiment, animals were acclimatized two weeks before the experiment and kept under standard environmental and nutritional conditions. Rats were kept in individual cages and fasted overnight prior to UC induction. Dapagliflozin was administrated orally (1 mg/kg, orally) for 14 days against AA-induced UC (2 mL, 3% *v*/*v*, intracolonic). Animals were divided into four experimental groups as follows: Normal control group: rats received vehicle orally once daily; DAPA group: rats received Dapagliflozin (1 mg/kg, orally); AA group, where the colitis was induced through intrarectal administration of AA and the rats received the vehicle once daily, orally, 48 h following induction of colitis; treated group (AA/Dapagliflozin): rats received DAPA (1 mg/kg, orally) for 14 consecutive days which started 48 h post-induction of UC.

### 2.4. Induction of Colitis

Animals fasted one night before induction of colitis, but they had free access to clean water. For the induction, animals were anesthetized with thiopental sodium (20 mg/kg. i.p). Later on, a polypropylene tube (2 mm) was gently and carefully inserted into the colon via the rectum at a distance of 8 cm, and then 2 mL of AA in normal saline (3% *v*/*v*) was slowly injected into colon. Rats were then retained for 30 s in a supine Trendelenburg position to avoid intracolonic instillation.

### 2.5. Scarification and Biological Samples Collection

The experimental animals were anesthetized with thiopental sodium (50 mg/kg) and sacrificed at the end of the experimental period. Blood samples were collected from the retro-orbital plexus and serum samples were obtained by centrifugation of blood at 4000 R.P.M. for 10 min. Sera were separated and kept in clean tubes, and stored at −20 °C until use for determination of the activity of lactate dehydrogenase (LDH), C-reactive protein (CRP), total antioxidant capacity (TAC), and malondialdehyde (MDA). 

Colon lengths and body weights were measured. Furthermore, colon weight/colon length ratio and colon/body weight index were calculated. Later on, colons were removed, excised from adherent adipose tissue, washed with normal saline (cold), and examined for macroscopic scoring. A longitudinal colon section was used to make colon homogenate for determination of colon IL-18, MCP-1, and inflammasome. Another section was also immersed in 10% neutral buffered formalin (NBF) for the following steps of histopathological examination and immunohistochemical analysis.

### 2.6. Clinical Evaluation of UC

The clinical evaluation of AA-induced colitis was determined though examination of stool consistency. Assessment of loose stool and diarrhea, occult and/or gross bleeding, hemoccult positivity, and body weight were the parameters to consider. The change in body weight for each rat was assessed, calculated, and compared to its corresponding weight before induction of UC using AA. This former step was performed to measure the disease activity index of AA colitis, which can be calculated by summing the weight loss, bleeding scores, and stool consistency, then dividing the result by 3 [21]. 

### 2.7. Macroscopic Examination and Grading of UC 

Following scarification and postmortem laparotomy, around 6 cm of colon extending approximately 2 cm above the anal margin was harvested and slit lengthwise, and the macroscopic changes in the colonic mucosa were scaled using a scoring system ranging from 0 to 4 as described elsewhere [22].

### 2.8. Preparation of Colon Homogenate 

Colon was homogenized in 1.15% KCl (ice-cold, pH 7.4), following the method described elsewhere [23], in order to produce 10% *w*/*v* colon homogenate. Homogenized tissues were then centrifuged at 4000 R.P.M. for 30 min (4 °C) and the supernatants were used for evaluation of the biochemical parameters.

### 2.9. Measurement of Serum Lactate Dehydrogenase (LDH) Activity and C-Reactive Protein (CRP) 

LDH levels were measured using the commercially available kit (Biomed Diagnostics assay kits, Cairo, Egypt) following the manufacturer’s protocol. On the other hand, CRP serum was measured using CRP-Latex (Chemelex, Spain) according the manufacturer instructions.

### 2.10. Determination of Oxidant/Antioxidant Stress Biomarkers: Malondialdehyde (MDA) and Total Antioxidant Capacity (TAC) Contents

Serum MDA and TAC were measured using colorimetric MDA and TAC kits (Biodiagnostic, Giza, Egypt) according to the manufacturer’s instructions and as described elsewhere [24]. 

### 2.11. Determination of Rat IL-18, MCP-1, and Inflammasome in Colon Homogenate

The concentrations of IL-18, MCP-1, and inflammasome in colon homogenates were quantified using Sandwich enzyme-linked immunosorbent assay (ELISA) kits (Bender MedSystems, Vienna, Austria). Briefly, 50 µL of assay diluent and 50 µL of control or sample were added per well in a 96-well microplate pre-coated with a monoclonal antibody specific for rat IL-18, MCP-1, and inflammasome. Rat IL-18, MCP-1, and inflammasome standards were used to construct the standard curve. After incubation for 2 h at room temperature, wells were washed and 100 µL of rat IL-18, MCP-1, and inflammasome conjugate was added to each well. Incubation of plates was performed for 2 h and the plate was washed, then 100 µL of substrate solution was added to each well, incubated for 30 min at room temperature, and protected from light. Finally, 100 µL of stop solution was added to each well and the optical density of each well was determined within 30 min, using a microplate reader set to 450 nm. 

### 2.12. Histopathological Examination and Immunohistochemical Evaluation of NF-κB and Caspase-3 Expression

Colon samples were fixed in a 10% NBF (pH 7.4). Following fixation of the samples, they were dehydrated in alcohol, cleared in xylene, and then embedded in paraffin. Tissue sections were then collected through microtome sectioning, and finally, the slides were prepared and stained with hematoxylin and eosin (H&E) for light microscopy. Scoring of the histopathological findings was performed according to a four-point score depending on the assessment of mucosal necrosis and ulceration, submucosal oedema, hemorrhage, and inflammation in eight HPFs as described elsewhere [25]. For immunohistochemical staining, paraffin sections were adhered on coated slides, cleared in xylene, rehydrated, and then immersed in an antigen retrieval (EDTA solution, pH 8). To remove nonspecific staining, the slides were incubated in 0.3% H2O2 and blocked in 5% bovine serum albumin in Tris-buffered saline (TBS) for 2 h. The slides were prepared and stained with anti-NF-κB P65) and Caspase-3 (Thermo Fisher Scientific, Waltham, MA, USA then washed with PBS for three successive times (20 min each), incubated with secondary antibody (EnVision + System HRP; Dako, Santa Clara, CA, USA) for 30 min at room temperature, then washed and incubated for 2 min in diaminobenzidine (DAB; Dako, Santa Clara, CA, USA). Subsequently, the slides counterstained with Mayer’s hematoxylin stain were covered with a glass cover slide. The labelling index of both NF-κB P65 and Caspase-3 were expressed as the percentage of cells (positive) per total 1000 counted cells using eight high-power fields [25].

### 2.13. Statistical Analysis

In each experimental group, data are expressed as means. SEM and ANOVA followed by the Tukey–Kramer multiple comparison test were used to evaluate the statistical significance of the results. The Kruskal–Wallis test, followed by Dunn’s Multiple Comparison test, was also used to examine the results of histopathology scoring. The Instat-3 and prism computer programs were used to conduct statistical testing (GraphPad Software Inc. V5., San Diego, CA, USA). The level of significance was considered at (*p* ˂ 0.05).

## 3. Results

### 3.1. DAPA Effects on AA-Induced Changes in Colon/Body Weight Index and Colon Weight/Colon Length Ratio

**Figure 1:**
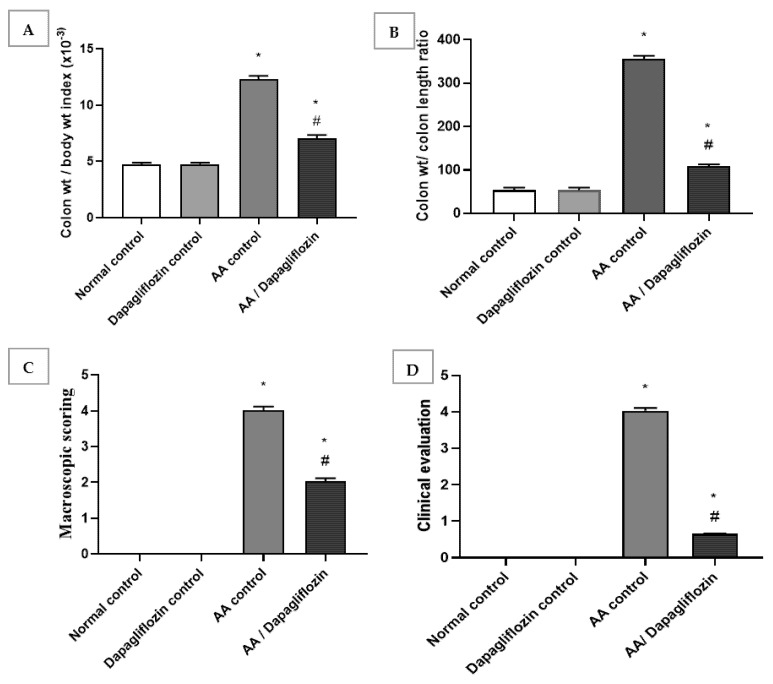
Effect of DAPA on AA-induced change in (**A**) Colon/body weight index, (**B**) Colon weight/colon length ratio, (**C**) Macroscopic scoring, and (**D**) cCinical evaluation. Data are expressed as mean ± SEM. Superscript characters reveal significant differences determined using one-way analysis of variance (ANOVA) followed by the Tukey–Kramer multiple comparison test (* *p* ˂ 0.05, DAPA compared with the normal control group; # *p* ˂ 0.05, DAPA compared with AA control group).

Normal rats treated with DAPA showed no relevant differences in colon/body weight index and colon weight/colon length ratio when compared with normal group. The colon/body weight index of AA was significantly increased by approximately 6.6-fold when compared to the normal group (*p* < 0.05). Treatment showed a significant decline in colon/body weight index by 69.3% versus the AA control group (*p* < 0.05). Likewise, there was a significant increase in colon weight/colon length ratio in AA control group by 2.6-fold in comparison to normal group. A remarkable decrease was revealed after treatment with DAPA by 43% when compared with AA control group (*p* < 0.05). However, it could not reach the level of the normal control group (Figure 1A,B).

### 3.2. Effect of DAPA on AA-Induced Changes in Macroscopic Scoring and Clinical Evaluation of Ulcerative Colitis

The obtained data revealed no significant change in macroscopic scoring and clinical evaluation between the normal control group and DAPA control group. Following intracolonic AA installation, macroscopic scoring and clinical evaluation of ulcerative colitis were each raised four-fold in comparison with the normal group (control). On the other hand, treatment with DAPA revealed a significant decrease in macroscopic scoring, as it inhibited macroscopic scoring by 49.68% compared to AA control group (*p* < 0.05). Treatment with DAPA inhibited the clinical evaluation of ulcerative colitis by 83.87% in comparison with AA control group (Figure 1C,D). 

### 3.3. Effect of DAPA on AA-Induced Alterations in Serum LDH, MDA, C-Reactive Protein, and Total Antioxidant Capacity

**Figure 2:**
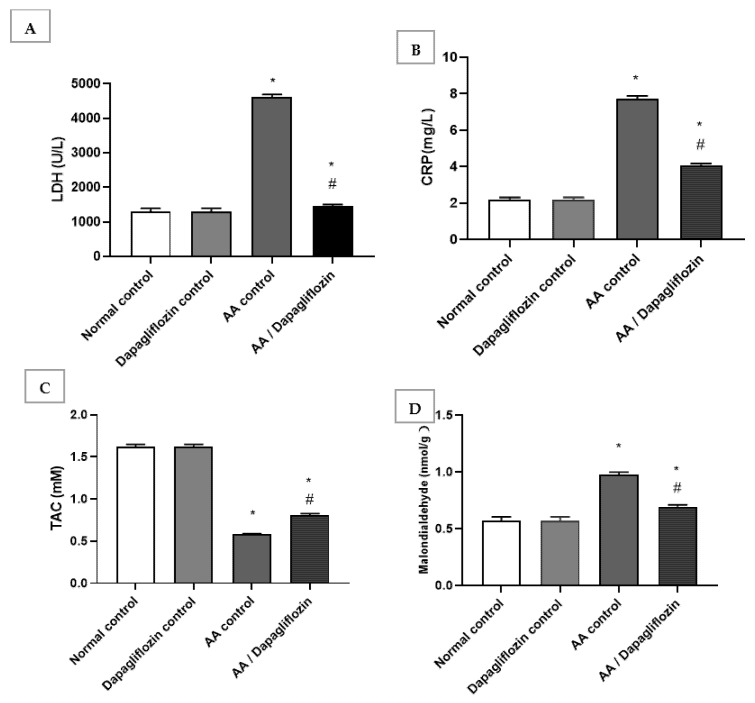
Effect of DAPA on AA-induced change in (**A**) serum Lactate dehydrogenase(LDH), (**B**) C-reactive protein(CRP), (**C**) Total antioxidant capcity(TAC), and (**D**) Malondialdehde(MDA). Data are expressed as mean ± SEM. Superscript characters reveal significant differences determined using one-way ANOVA followed by the Tukey–Kramer multiple comparison test (* *p* ˂ 0.05, DAPA significantly different from the normal control group; # *p* ˂ 0.05, DAPA significantly different from AA control group).

The normal group treated with DAPA revealed no significant changes in serum LDH, C-reactive protein, MDA, or total antioxidant capacity compared to the normal control group. In this respect, serum LDH and CRP increased by 3.53-fold and serum by 6.62-fold, respectively, in the AA control group versus normal control group (*p* < 0.05). Furthermore, treatment with DAPA resulted in a significant decrease in serum LDH activity (*p* < 0.05) by 68.53% compared to AA control group (Figure 2A). On the other hand, the LDH level was significantly decreased in the UC group treated with DAPA by 47.07% versus AA control (*p* < 0.05). However, it did not reach the normal level in the control group. Moreover, the intracolonic installation of AA resulted in an approximately 0.35-fold decrease in serum TAC; meanwhile, there was marked increase in MDA by approximately 1.7-fold compared with normal group (control). On the other hand, treatment of UC with DAPA resulted in a significant increase (*p* < 0.05) in TAC level by 39% versus the AA control group (Figure 2C). Furthermore, treatment with DAPA resulted in a significant decrease (*p* < 0.05) in MDA level by 28.9% compared to the AA control group, but this value was still significantly different to that of the normal control group (Figure 2D). 

### 3.4. Effect of DAPA on AA-Induced Changes in Colon IL-18 Content

**Figure 3:**
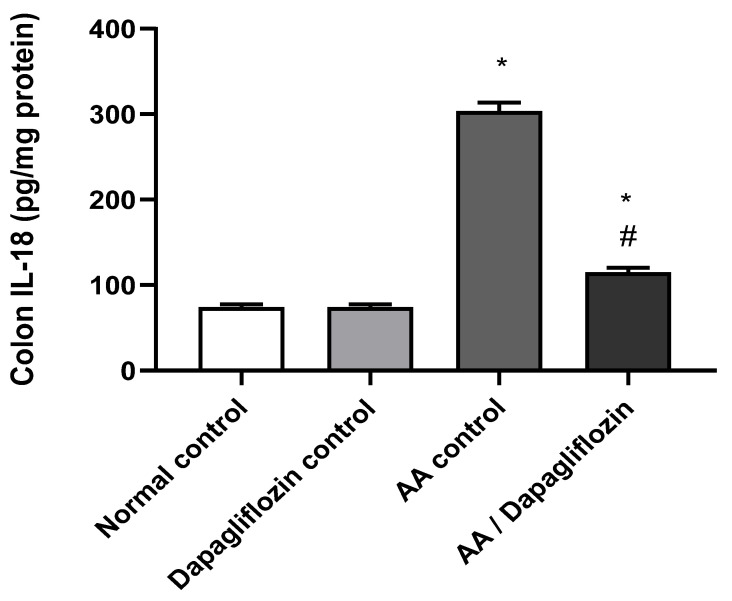
Effect of DAPA on AA-induced change in Interleukin- 18( IL-18) in colon tissue. Data are expressed as mean ± SEM. Superscript letters indicate the level of statistical significance that was performed using one-way ANOVA followed by the Tukey–Kramer multiple comparison test (* *p* ˂ 0.05, DAPA significantly different from the normal control group, ^#^ *p* ˂ 0.05, DAPA significantly different from the AA control group).

IL-18 is considered a pro-inflammatory signaling pathway. As shown in Figure 3, no significant difference was observed in colon IL-18 content between the normal control group and the DAPA control group. Following AA installation, a significant increase (four-fold) was noticed in colon IL-18 content compared to the normal control group (*p* < 0.05). Moreover, treatment of UC with DAPA resulted in a significant down-regulation of colon IL-18 by 55% content versus the AA control group (*p* < 0.05), but it did not reach the level of the normal control group (Figure 3).

### 3.5. Effect of DAPA on AA-Induced Changes in Colon MCP1 Content

**Figure 4:**
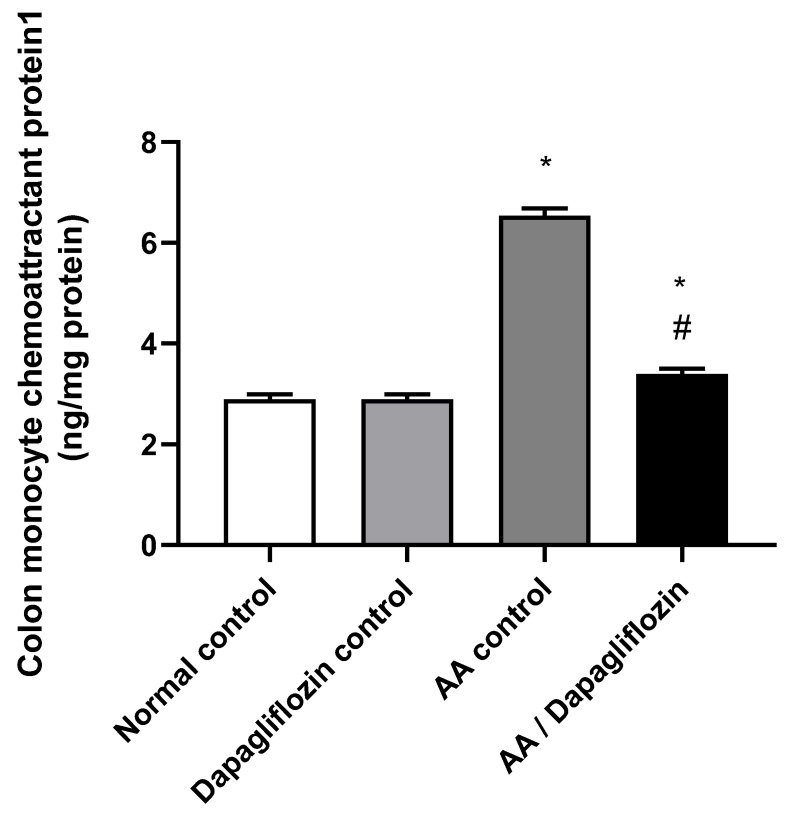
Effect of DAPA on AA-induced change in Monocyte chemoattractant protein(MCP1) in colon tissue. Data are expressed as mean ± SEM. Statistical analysis was performed using one-way ANOVA followed by the Tukey–Kramer multiple comparison test (*p* ˂ 0.05). Superscript letters refer to the level of significance: * DAPA significantly different from the normal control group; # DAPA significantly different from the AA control group).

MCP1 is a signaling pathway that promotes inflammation. As depicted in Figure 4, no significant changes were reported in colon MCP1 content between the normal control group and DAPA control group. On the other hand, AA installation resulted in a significant increase in colon MCP1 content by 2.25-fold compared to normal group (*p* < 0.05). Furthermore, administration of DAPA has resulted in a remarkable decrease in the colon MCP1 content by 47.95% versus the AA control group (*p* < 0.05), while the value was still significantly different from the normal control group (*p* < 0.05).

### 3.6. Effect of DAPA on AA-Induced Changes in Colon Inflammasome Content

**Figure 5:**
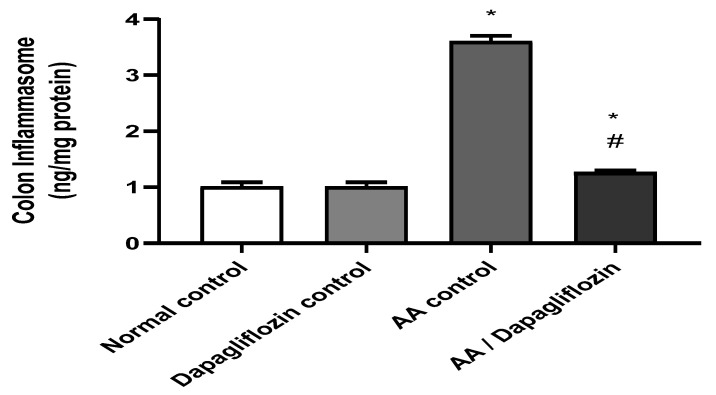
Effect of DAPA on AA-induced change in inflammasome in colon tissue. Data are expressed as mean ± SEM. Statistical analysis was performed using one-way ANOVA followed by the Tukey–Kramer multiple comparison test (*p* ˂ 0.05). Superscript letters refer to the level of significance: * DAPA Significantly different from the normal control group; # DAPA significantly different from the AA control group).

Inflammasome is a signaling pathway associated with inflammation. Figure 5 shows that no significant difference was observed in colon inflammasome content between the normal control group and the DAPA control group. However, AA installation resulted in a significant increase in colon inflammasome content (3.57-fold) versus the normal control group (*p <* 0.05). On the other hand, treatment of UC with DAPA was accompanied by a significant decrease in colon inflammasome content of 64.80% in comparison with the AA control (*p <* 0.05). However, this value did not reach that of the normal group.

### 3.7. Effect of DAPA on AA-Induced N Histopathological Changes 

**Figure 6:**
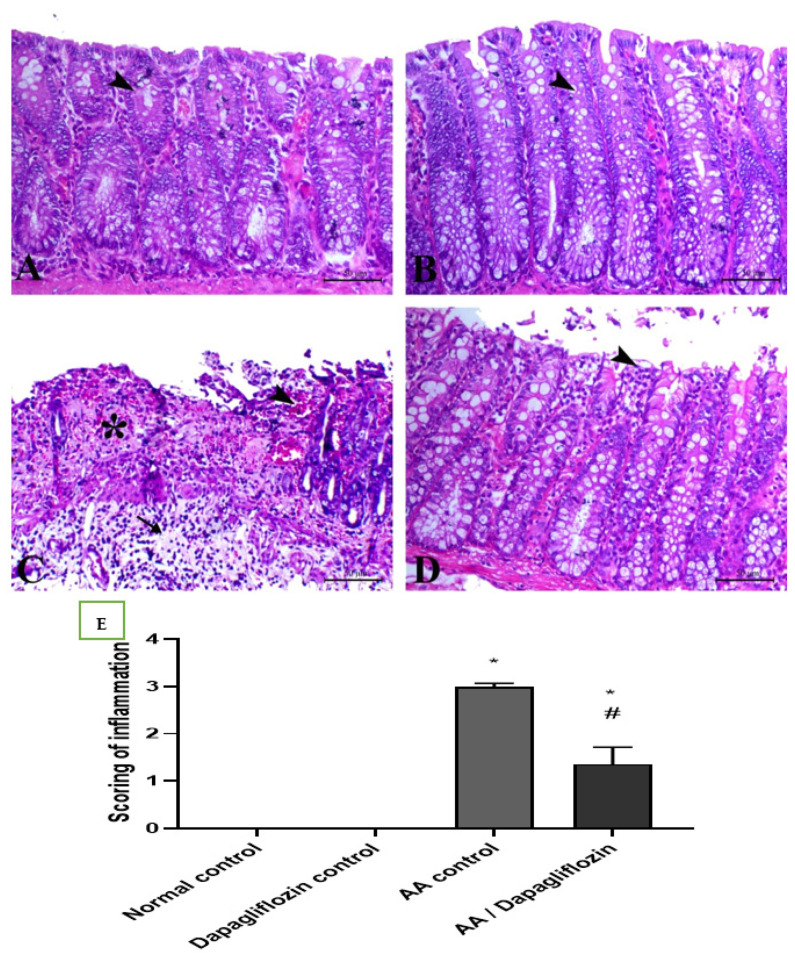
(**A**) Colonic sections stained by H&E showing the effect of DAPA for 14 days on AA-induced UC. Colonic sections from normal (**A**) and Dapagliflozin (**B**) control groups showing normal mucosa including surface epithelium and crypts (arrowheads). Colonic sections from the AA-induced colitis group (**C**) showing marked necrosis of the mucosal lining mixed with fibrin exudate and submucosal edema with marked inflammatory cells infiltration. Colonic sections from the Dapagliflozin-treated group (**D**) showing superficial mucosal necrosis (arrowhead). H&E, bar = 50 µm. (**E**) Scoring of inflammation. Data are expressed as mean ± SEM. Superscript letters refer to the level of statistical significance which was calculated using one-way ANOVA followed by the Tukey–Kramer multiple comparisons test (* *p* ˂ 0.05, DAPA compared to the normal control group; # *p* ˂ 0.05, DAPA significantly different from the AA control group).

Colon sections from the normal control group revealed normal colon architecture, normal mucosal crypts, and submucosal tissues with no evidence of inflammatory lesions. Similarly, the normal rats treated with DAPA showed normal colon tissue with an interesting elongation of intestinal crypts and noticeably increased goblet cells within their lining epithelium. Diseased rats treated with AA showed massive ulcerative hemorrhagic lesions associated with massive confluent necrosis along the mucosa, fibrin deposition within the mucosa and submucosa, and marked inflammatory cells infiltration along the mucosa and lamina propria and extending deep to the musculature. Treatment of UC with DAPA showed an improvement in mucosal lesions that were restricted to the epithelial covering, with marked decrease in the edema and fibrin deposition within the lamina propria, and marked decrease in inflammatory-cell infiltration (Figure 6A–D). The quantitative scores of histopathological lesions are shown in Figure 6E and Table 1, respectively.

### 3.8. Effect of DAPA on AA-Induced Changes in Colon Inflammation, Immunohistochemical Analysis of NF-κB Expression

**Figure 7:**
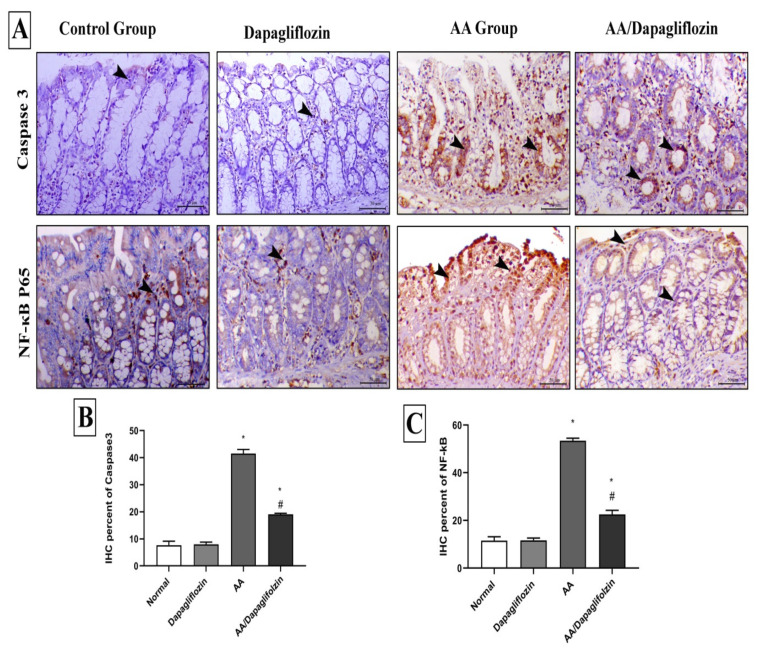
Immunohistochemical analysis of colonic expression of Caspase-3 and NF-κB P65 antibodies. (**A**) The first panel indicates the immunostaining of Caspase-3 within the colonic mucosa and the second panel indicates the expression of NF-κB P65 within the mucosa of the different treated groups (arrowheads indicate the positive expression). (**B**) Quantitative scoring of Caspase-3 represented with the mean of the percentage of positive expression of the different groups. (**C**) Quantitative scoring of NF-κB P65 represented with the mean of the percentage of positive expression of the different groups. Superscript letters indicate the level of statistical significance that was performed using one-way ANOVA followed by the Tukey–Kramer multiple comparisons test (* *p* ˂ 0.05, DAPA compared to the normal control group; # *p* ˂ 0.05, DAPA compared to the UC control group).

The control and sham groups showed mild immunostaining for NF-κB P65, which was mostly seen within the interstitial tissues. On the contrary, AA administration revealed a marked increase in the expression of nuclear NF-κB P65. Colonic specimens from diseased animals treated with DAPA also showed marked decrease in the expression of NF-κB P65 (Figure 7). 

### 3.9. Effect of DAPA on AA-Induced Changes in Colon Apoptosis, Immunohistochemical Analysis of Caspase-3 Expression

Caspase-3 immunostaining was seen in a scanty manner in the intestinal crypts of both control and sham animals. AA treatment demonstrated marked increase in both cytoplasmic and nuclear expression of Caspase-3 expression in colon tissue. Caspase-3 expression was markedly decreased in colonic tissues from diseased rats treated with DAPA (1 mg/kg) (Figure 7). 

## 4. Discussion

The current study investigated the potential therapeutic effects of Dapagliflozin against experimentally produced UC. The results of current study revealed that DAPA mitigated UC induced by acetic acid in a rat model. The oral administration of DAPA also showed an improvement in all measured biochemical parameters, and histopathological and immunohistochemical examination of colon specimens. Intracolonic administration of AA is a standard model for induction of UC [26]. In the present study, AA induced significant increase in macroscopic scoring of colitis by affecting stool consistency and inducing hemorrhage. The present observations are in line with a previous study [27], in which acetic acid caused inflammation in the colon of laboratory animals. Furthermore, intra-rectal injection of AA induced significant weight loss due to nutrient deficiency resulting from decreased appetite, malabsorption, and fast body-fluid loss resulting from colorectal bleeding. Taken into account, TNF-α and IL-6 play an important role in body-weight loss induced by the secretion of neuropeptides, suppressing the appetite in colitis [28]. In the present work, treatment of UC with DAPA significantly improved clinical evaluation and macroscopic scoring of colitis induced by AA and these results are consistent with previous results of a study that reported that DAPA prevented colon shortening and declined disease activity through targeting the NF-κB/AMPK/NLRP3 axis [29]. It seems that inflammation associated with UC contributed to the presence of oxidative stress in the colon with the production of reactive oxygen and nitrogen species (ROS, RNS). Generation of free radicals is directly associated with the degree of severity of UC. Likewise, oxidative stress plays a major role in the progression of chronic inflammation into colon cancer. This disturbed oxidative homeostasis is associated with deficient antioxidant mechanisms [30]. In the present study, AA injection into the colon significantly increased inflammation in colonic tissue, evidenced by elevated markers of inflammation such as LDH and CRP, and this effect is consistent with a previous study [31], which reported that 4% acetic acid caused overproduction of proinflammatory mediators, ROS, RNS, platelet-activating factor, eicosanoids, and cytokines. These mediators induce the extension of the inflammatory response of the gut as discussed in a previous study [32].

The current study also revealed that daily administration of oral DAPA for 14 days decreased inflammation and its markers (LDH, CRP). These results are consistent with a previous study [33], which revealed that 100 mg canagliflozin every other day improved endothelial function in diabetic patients with suspected coronary disease through decreased markers of inflammation. Furthermore, the present study reported that AA inducing marked oxidative stress with significantly elevated MDA and reduced TAC in colonic tissue, which is in harmony with a previous study [34]. In another study, intrarectal administration of 3% *v*/*v* AA increased colonic MDA and decreased colonic TAC due to increased production of oxidant radicals and generation of excessive ROS, which induced damage of biologic molecules of cellular components [35]. The group treated with DAPA in the recent study relieved the degree of oxidative stress induced by AA through decreasing the levels of colon MDA and increasing the level of TAC in colon. This finding is in agreement with a previous study [36], in which administration of DAPA by a dose of 0.1 mg/kg body weight once a day by oral gavage for diabetic rats managed oxidative stress and significantly decreased its markers and protected cardiovascular function in type-2 diabetics. This antioxidant and protective effect of DAPA occurred through inhibiting the activation of the death pathways involved in cell apoptosis and necrosis [37]. It is noteworthy to state that IL-18 is a potent proinflammatory cytokine which might induce colitis through the activation of inflammatory mediators such as TNF-α and chemokines [38]. Stimulation of p-NF-κB p65 initially activates NLRP3 inflammasome and Caspase-1 and stimulates IL-1β and IL-18, which in turn triggers the production of other inflammatory cytokines including IL-6 and TNF-α [39]. The current study showed that intrarectal administration of AA significantly elevated colon IL-18, which is consistent with the aforementioned findings of a previous study [40], in which intrarectal administration of AA increased colon level of IL-18 through upregulation of the TNFα/NF-κB/NLRP3 inflammatory pathway. This study reported that oral administration of DAPA for 14 days significantly declined IL-18 levels in colon tissues confirming former results of Leng et al., (2016) [41], in which intragastric administration of DAPA 1.0 mg/kg/day for 12 weeks decreased the production of IL-1β and IL-18 proteins in abdominal aorta of diabetic mice. This effect may be due to reduced production of ROS and inhibition of NLRP3 inflammasome activation in the aortic root of diabetic mice as discussed elsewhere [42]. It should be stressed that intestinal homeostasis and inflammation are also controlled by interleukin-1 (IL-1) cytokines [43]. IL-18 is activated by cysteine protease Caspase through its activation by inflammasome then binds to IL-18 receptor alpha chain, resulting in chemotaxis of IL-1 receptor-associated kinase (IRAK) and TNF receptor-associated factor (TRAF6), which in turn stimulate inhibitor of κB (IκB) kinases (IKKs), IKKα and IKKβ [44]. 

Monocyte chemotactic protein-1 (MCP-1) is considered a potent chemotactic cytokine which controls the chemotaxis and infiltration of monocytes/macrophages in the gut. It is released by dendritic cells in the mucosa to regulate inflammatory process in the site of tissue damage [45]. It is also produced by macrophages, endothelial cells, and fibroblasts, and its expression is stimulated after exposure to inflammatory stimuli such as TNF-α and IL-1 produced by Th1 cells in the colon [46]. Importantly, MCP-1 enhances the expression and production of other inflammatory cytokines, infiltration of inflammatory cells, and macrophages [47]. Our results showed that colonic levels of MCP-1 in the acetic acid group were significantly increased, which is in line with the previous studies of [48], which reported that intrarectal administration of 2 mL 4% AA upregulated the expression of levels of MCP-1 gene in colon tissues, illustrating the proinflammatory effect of AA. In contrast, treatment of rats with DAPA induced significant reduction in this proinflammatory cytokine, MCP-1, which is in harmony with a previous study [49], which showed that 10 or 100 µM DAPA declined mRNA and protein expression of MCP-1 in human proximal tubular cell line, and in turn demonstrated the anti-inflammatory effect of DAPA through downregulation of high-mobility group box 1 (HMGB1)-receptor for the advanced glycation end-products (RAGE)-NF-κB pathway. A previous study [10] reported that mice deficient in MCP-1 gene showed less DNBS-induced colonic damage both macroscopically and histologically with a smaller number of CD3^+^ T cells, 5-HT-expressing EC cells, and F4/80^+^ macrophages in addition to decreased activity of IL-12, IFN-γ, and il-1β in comparison with wild-type (MCP-1^+/+^) mice, which illustrated the essential role of MCP-1 in the pathogenesis of inflammation. In addition, NF-κB controls the expression of inflammatory mediators, including TLR4, following stimulation by the inflammatory insult [50].

The current study also revealed that the group which received AA experienced elevated levels of inflammasome in colon tissues, which is consistent with a previous study [51] which revealed that intracolonic administration of AA elevated the colonic expression of inflammasome. The group of rats treated with DAPA had significantly reduced colonic levels of inflammasome in comparison with acetic acid group and these results agree with a prior study [52] which reported that intragastric administration of 1.0 mg/kg/d DAPA for 12 weeks diminished the activity of hepatic inflammasome in an experimental model of diabetic steatohepatitis due to the antioxidant effect of DAPA and suppressed mitochondrial production of ROS. In the present work, oral administration of DAPA succeeded in decreasing scores of mucosal ulceration, necrosis, hemorrhage, edema, and inflammation induced by AA after histopathological examination of colon specimens, besides decreasing the degree of NF-κB expression within the colon after immunohistochemical analysis. These results are consistent with the previous findings of Lee et al., 2020 [53], which reported that administration of (1mg/kg/day) DAPA for 8 weeks reduced inflammatory processes in the milieu of the atherosclerotic plaques of rabbits through decreasing the immunostaining of NF-κB in the DAPA-treated group in the aorta wall vascular cells of the rabbits due to an indirect immunomodulatory effect via regulation of the IκB kinase (IKK)/IκB/NF-κB signaling pathway, besides its direct link with the TLR4 signaling pathway [54]. 

In the present study, treatment with DAPA revealed a significant decrease in Caspase-3 expression. Previous studies reported that empagliflozin prevented beta-cell death by reducing glucotoxicity-induced oxidative stress [55]. The same study hypothesized that hyperglycemia-induced oxidative stress has an upstream influence on apoptosis, and that reduction in blood glucose through SGLT2 inhibition lowered apoptosis and enhanced the beta-cell mass in diabetic rats [56]. Clearly, this finding established that GLT2 inhibition, using DAPA, might prevent obesity induced apoptosis by attenuation of oxidative stress, ER stress, and obesity in renal tissues of diabetic rats [57]. Another study found that DAPA reduced apoptotic processes in diabetic rats’ renal tissue by lowering oxidative stress and reducing renin–angiotensin system (RAS) activity [58]. Furthermore, in HK2 cells treated with high glucose concentrations, SGLT2 inhibition by empagliflozin protected renal proximal tubular cells from death by lowering intra-renal lipo-toxicity. They also released that empagliflozin increased Bcl-2 expression, decreased Bax and cytochrome-C protein expression, and inactivated Caspase-3,8, and 9 in kidney tissues [56]. Another study reported the anti-apoptotic potentials of SGLT2 in the cardiovascular network, which could be attributed to cardio-protective effects of these anti-diabetic medicines [59].

## 5. Conclusions

Collectively, Dapagliflozin provided anti-ulcerogenic and colo-protective effects against experimentally induced UC in rats. The therapeutic impact is primarily mediated via downregulation of MCP1, IL-18 signaling, and NF-κB expression. Antioxidant, anti-inflammatory, and anti-apoptotic characteristics of Dapagliflozin interplay in its anti-ulcerogenic and colo-protective impact. Elucidation of new mechanistic pathways in ulcerative colitis is also suggested. 

## Figures and Tables

**Table 1:** Effect of DAPA on AA-induced change in count of inflammatory cells for H&E.

	Normal Control	Dapagliflozin Control	AA Control	Dapagliflozin
Mucosal ulceration	−	−	+++	−
Mucosal necrosis	−	−	+++	++
Hemorrhage	−	−	++	+
Edema	−	−	+++	++
Inflammation	−	−	+++	−

Score or degree of lesions; (− negative; + = mild; ++ = moderate; +++ = severe).

## Data Availability

The data that support the findings of this study are available on request from the corresponding author.
